# Identification and expression analysis of *GRAS* transcription factors in the wild relative of sweet potato *Ipomoea trifida*

**DOI:** 10.1186/s12864-019-6316-7

**Published:** 2019-11-29

**Authors:** Yao Chen, Panpan Zhu, Shaoyuan Wu, Yan Lu, Jian Sun, Qinghe Cao, Zongyun Li, Tao Xu

**Affiliations:** 1 Key lab of phylogeny and comparative genomics of the Jiangsu province, Jiangsu Normal University, Xuzhou, Jiangsu Province, 221116 China; 2 Department of Plant Biotechnology, College of Agriculture and Life Sciences, Chonnam National University, Gwangju, 500-757 South Korea; 3 Xuzhou Academy of Agricultural Sciences/Sweet Potato Research Institute, Xuzhou, 221121 Jiangsu China; 4 Department of Organismic and Evolutionary Biology, Harvard University, Cambridge, MA 02138 USA

**Keywords:** GRAS, Transcription factor, Sweet potato, *Ipomoea trifida*, Expression

## Abstract

**Background:**

*GRAS* gene is an important transcription factor gene family that plays a crucial role in plant growth, development, adaptation to adverse environmental condition. Sweet potato is an important food, vegetable, industrial raw material, and biofuel crop in the world, which plays an essential role in food security in China*.* However, the function of sweet potato *GRAS* genes remains unknown.

**Results:**

In this study, we identified and characterised 70 GRAS members from *Ipomoea trifida*, which is the progenitor of sweet potato. The chromosome distribution, phylogenetic tree, exon-intron structure and expression profiles were analysed. The distribution map showed that *GRAS* genes were randomly located in 15 chromosomes. In combination with phylogenetic analysis and previous reports in *Arabidopsis* and rice, the GRAS proteins from *I. trifida* were divided into 11 subfamilies. Gene structure showed that most of the *GRAS* genes in *I. trifida* lacked introns. The tissue-specific expression patterns and the patterns under abiotic stresses of *ItfGRAS* genes were investigated via RNA-seq and further tested by RT-qPCR. Results indicated the potential functions of ItfGRAS during plant development and stress responses.

**Conclusions:**

Our findings will further facilitate the functional study of *GRAS* gene and molecular breeding of sweet potato.

## Background

GRAS proteins are a family of plant-specific transcription factors whose names are derived from the first three members: GIBBERELLIN ACID INSENSITIVE (GAI), REPRESSOR of GA1 (RGA) and SCARECROW (SCR) [1]. Typically, GRAS proteins consist of 400–770 amino acids residues with a variable N-terminal and a highly conserved C-terminal region [2, 3]. The highly conserved carboxyl terminal region is composed of several ordered motifs, including leucine rich region I, VHIID, leucine-rich region II, PFYRE and SAW, which are crucial for the interactions between GRAS and other proteins [1, 4]. According to the report in *Arabidopsis thaliana*, the GRAS family is classed into eight well-known subfamilies, including LISCL, PAT1, SCL3, DELLA, SCR, SHR, LAS and HAM [5]. However, Liu et al. (2014) classified the *GRAS* family into 13 branches. The subfamily identification of *GRAS* genes has a slight difference among diverse species.

In the recent 10 years, with increasing species having complete genome sequence, the genome-wide analyses of *GRAS* gene family were carried out in more than 30 species belonging to more than 20 genera, such as in *A. thaliana* [4], rice [4], maize [6], Chinese cabbage [7], tomato [8], *Prunus mume* [9] and *Poplar* [10]. GRAS proteins play diverse functions in regulating plant growth and development, which are involved in signal transduction, root radial patterning [11], male gametogenesis [12] and meristem maintenance [2]. *GRAS* genes are connected with plant disease resistance and abiotic stress response [13]. *OsGRAS23* enhances tolerance to drought stress in rice [14]. The overexpression of poplar *PeSCL7* in *Arabidopsis* increases its resistance to drought and salt stresses [15]. Likewise, Yang et al. (2011) reported that the overexpression *BnLAS* gene of *Brassica napus* in *Arabidopsis* can enhance the drought tolerance of plant [16]. DELLA proteins are involved in response to adverse environmental conditions such as low temperature and phosphorus deficiency [17, 18]. Moreover, *NtGRAS1* in tobacco increases the ROS level under various stress conditions [19]. Although these genes play critical roles during plant growth, development and abiotic stress adaption, *GRAS* gene has not been studied in sweet potato and the other *Ipomoea* plant.

Sweet potato *[Ipomoea batatas (L.) Lam.]* is an important food crop, which ranks seventh in the world [20]. Due to its rich carbohydrates, dietary fibre, vitamins and low input requirements, it is widely grown in tropical areas, especially in sub-Saharan Africa. Recently, a comprehensive phylogenetic study of all species closely related to the sweet potato was presented and strongly supported nuclear and chloroplast phylogenies demonstrating that *Ipomoea trifida* (Kunth.) G. Don (2*n* = 2*x* = 30) is the closest relative of sweet potato [21]. And *I. trifida* is one of the most important material for studying self-incompatibility, sweet potato breeding, sweet potato transgenic system construction and whole genome sequencing due to its small size, low ploidy, small chromosome number and simple genetic manipulation [21–23]. In 2017, the genome data of *I. trifida* were released (http://sweetpotato.plantbiology.msu.edu/), thus allowing the genome-wide identification and analysis of important gene families in *I. trifida* [23]*.*

Therefore, we performed the genome-wide identification of GRAS transcription factors in *I. trifida.* We firstly investigated the phylogeny, chromosomal locations and exon/intron structure of GRAS transcription factors in *I. trifida*. Moreover, we checked the expression profiles of *ItfGRAS* genes in different tissue under various abiotic stress conditions by analysing RNA-Seq data and qRT-PCR experiment validation. Our work will provide evidence for further study of *GRAS* gene function and sweet potato breeding.

## Methods

### Identification of *GRAS* genes in *I. trifida*

All candidate *ItfGRAS* genes were derived from Sweetpotato Genomics Resource (http://sweetpotato.plantbiology.msu.edu/index.shtml). The Pfam database (http://pfam.xfam.org/search) was used to identify all likely GRAS proteins containing GRAS domains. To further confirm amino acid sequences with GRAS domains, we used the NCBI Conserved Domain search and SMART to ensure the accuracy of these transcription factors. Only the sequences with full-length GRAS domain were used for further analyses. At the same time, the online software ExPASy (http://expasy.org/tools/) was used to obtain the molecular weight (MW), isoelectric point (pI) and amino acid numbers of ItfGRAS proteins. We predicted the subcellular locations of these GRAS proteins by using the online WoLF PSORT (http://wolfpsort.org/).

### Chromosomal location and exon–intron structures analysis of *GRAS* members in *I. trifida*

The physical positions of all *ItfGRAS* genes were determined using GFF annotation file downloaded from Genomic Tools for Sweetpotato Improvement (GT4SP) project. We mapped the genetic linkage map of *GRAS* genes in the whole *I. trifida* genome by using MapDraw.

The web-based bioinformatics tool GSDS 2.0 (gsds.cbi.pku.edu.cn/) [24] was used to identify information on the intron/exon structure by comparing the coding domain sequences and genomic sequences of *ItfGRAS* genes.

### Phylogenetic analysis of GRAS proteins

We obtained *Arabidopsis* and rice GRAS amino acid sequences from plant TFDB (http://planttfdb.cbi.pku.edu.cn/). *I. trifida* GRAS proteins were aligned with the well-classified *Arabidopsis* rice GRAS proteins by using ClustalW to generate a phylogenetic tree. The phylogenetic analysis of the aligned sequences was then carried out by using the Maximum-Likelihood method. As a tool for building a phylogenetic tree, MEGA 7.0 [25] has parameters set to the P-distance model and pairwise deletion options with 1000 bootstrap replicates. During this construction, several ItfGRAS proteins with relatively less amino acid residues than the amino acid residues in typical GRAS domain were excluded.

### Analysis of *Cis*-acting elements in *ItfGRAS* promoters

To determine *cis*-acting elements in the promoter regions of *ItfGRASs*, we first extracted the promoter sequences (2 kb) for every *ItfGRAS* gene from *I. trifida* genomic DNA, and then submitted the sequences to online tool PlantCARE (http://bioinformatics.psb.ugent.be/webtools/plantcare/html/) [26] to predict *cis*-acting elements in *ItfGRAS* promoters. And TBtools software (v0.6654) (https://github.com/CJ-Chen/TBtools) was used to visualize the final results.

### Expression analysis of GRAS members

We downloaded the original RNA sequencing data from the GT4SP Project Download page to investigate the expression profiles of *GRAS* genes under abiotic stresses (drought, salt, heat and cold) and among various tissues (root, stem, leaf, flower and flower bud). Heat maps and hierarchical clustering for *I. trifida GRAS* genes based on fragments per kilobase million (FPKM) values were generated using MeV v4.8.1 [27]. The expressions of *ItfGRASs* in various tissues were normalized by Z-score, and all FPKM values of tissue specific expression and abiotic stresses are shown in Additional file 5-6: Table S4-S5.

### Plant materials and stress treatments

*I. trifida* (2*x*) plants were collected from the Sweet Potato Research Institute, Xuzhou Academy of Agricultural Sciences, National Sweet Potato Industry System, China. *I. trifida* growing up to 4 weeks was used as experimental material in this study. The growth conditions of *I. trifida* were as follows: light/dark for 16/8 h at 28 °C day/ 22 °C night.

For cold treatment, the 4-week-old *I. trifida* was transferred into a light incubator at 10 °C. Under heat treatment, these plants were grown in a light incubator at 39 °C. A 250 mM NaCl was poured into the pots under salt treatment. For drought treatment, whole plants were perfused with 300 mM mannitol solution. For the above treatments, all plants were grown under a 16/8 h (light/dark) photoperiod. Each treatment group was set to a control (without any treatment, growing under normal conditions). Leaf and root samples for experiment were obtained at 0, 6, 12, 24 and 48 h after treatment. All samples were frozen in liquid nitrogen and stored at − 80 °C for subsequent use.

### RNA isolation and qRT-PCR analysis

To validate the data of expression patterns based on RNA sequencing, we selected 10 genes with significantly high expression levels under stress and among tissues. The samples collected above include root, stem, mature leaf, young leaf and flower for tissue-specific expression and root and leaf samples for abiotic stresses. Total RNA was extracted from the frozen samples by using an RNAprep pure plant kit (TIANGEN, Beijing, China). The PrimeScript™ RT Reagent Kit (Takara, Dalian, China) was used to synthesize the first-strand complementary DNA (cDNA) with 1 μg of total RNA in a 20 μL volume according to the manufacturer’s protocols. The specific GRAS primers for qRT-PCR analysis were designed using Primer Premier 5 and are shown in Additional file 7: Table S6. The *GAPDH* gene was used as internal control gene. qRT-PCR analysis was performed using an ABI StepOnePlus instrument and the SYBR premix Ex Taq™ kit (TaKaRa, China). The thermal circulation conditions were set as follows: 95 °C for 5 min, 95 °C for 10 s and 60 °C for 20 s followed by 40 cycles. The specificity of each primer pair was verified by melting curve analysis. We analysed the expression profiles by calculating the mean of the expression levels obtained from three independent experiments according to the 2 − ^ΔΔCt^ method reported by Livak et al. (2001) [28].

### Statistical analysis

The qRT-PCR raw data were calculated according to the 2 − ^ΔΔCt^ method [28], and then subjected to ANOVA and means compared by the Dunnett’s test (“*” for *P* < 0.05). The SPSS software package (v.22) was used for statistical analysis. Microsoft Excel 2010 was used to calculate the standard errors (SEs). Graphpad prism 5.0 software was used to generate graphs.

## Results

### Identification and characterization analysis of *GRAS* genes in *I. trifida*

To identify the number of GRAS members in *I. trifida*, we used both Pfam and SMART databases with the default parameters. A total of 75 candidate *ItfGRAS* genes were identified. Among them, five *ItfGRAS* genes were excluded, because the GRAS domain region in those proteins contains less amino acid residues than the typical GRAS domain (Additional file 2: Table S1). Hence, only 70 *ItfGRAS* genes were finally kept and used for further analyses, and the result of 70 ItfGRAS protein sequence alignments are shown in Additional file 1: Fig. S1. Basic information, such as the number of amino acids, MWs, theoretical pI and intron numbers, for the GRAS proteins in *I. trifida* is listed in Additional file 2: Table S1. The length and MW/kDa of 70 GRAS proteins were 178–957 aa and 20–103.9 kDa, respectively. The predicted pI of *I. trifida* ranged from 4.76–9.45 (Additional file 2: Table S1).

### Chromosomal distributions of *ItfGRAS* genes

**Fig. 1:**
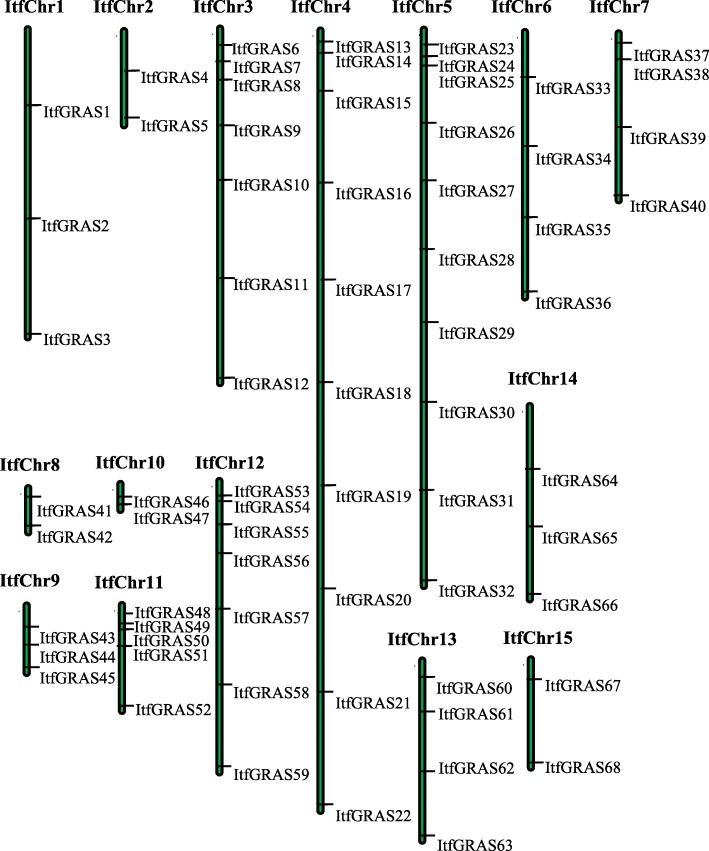
Chromosomal locations of *GRAS* genes in *I. trifida* along 15 chromosomes

The identified *GRAS* genes were mapped to 15 *I. trifida* chromosomes according to the download GFF3 profile. However, two GRAS members were not obviously mapped onto any chromosomes but were located on unattributed scaffolds. *ItfGRAS* genes were unevenly distributed among chromosomes. Figure 1 shows that Chr4 and Chr5 containing 10 (14.7%) GRAS members were the most abundant. Chr2, Chr8, Chr10 and Chr15 contained only two genes (3%), while the number of genes located in the remaining chromosomes ranged from 3 to 7.

### Evolutionary relationships of *GRAS* genes among three species

**Fig. 2:**
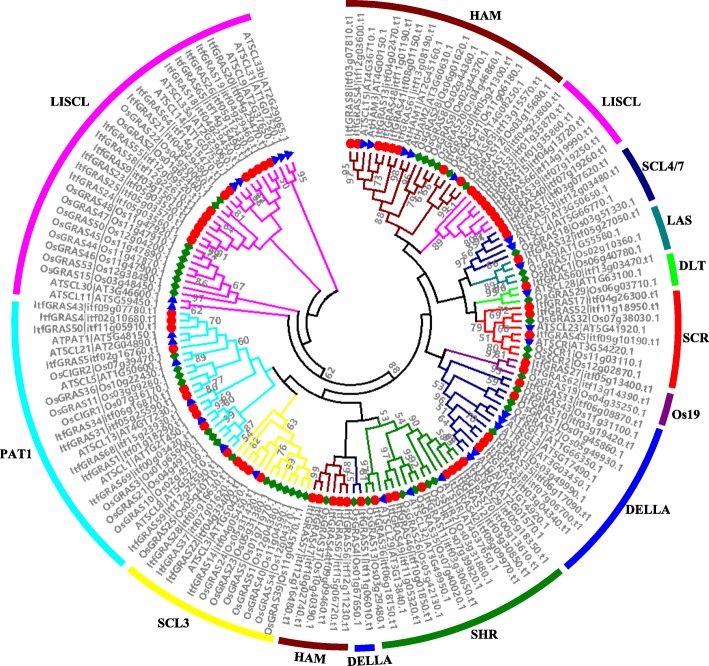
Phylogenetic analysis of GRAS proteins in *Arabidopsis*, *Oryza sativa L* and *I. trifida*. A phylogenetic tree of all the identified GRAS proteins among three species was constructed using MEGA 7.0 by the Maximum-Likelihood method analysis with 1000 bootstrap replications. The tree was classified into 11 different subfamilies indicated by different colored branches and outer rings. The red solid circles indicate the *I. trifida* GRAS proteins, the green solid diamonds represent the *Arabidopsis* GRAS proteins, and the blue solid triangles represent the *O. sativa* GRAS proteins. The bootstrap values > 50% are shown

To investigate the GRAS protein evolutionary relationship between *I. trifida* and the other known species, we constructed a phylogenetic tree containing 70 GRAS proteins from *I. trifida*, 50 GRAS proteins from *Oryza sativa* and 33 proteins from *Arabidopsis* (Additional file 3: Table S2). Figure 2 showed us that the ItfGRAS proteins were classified into 11 subfamilies, namely, HAM, DELLA, SCL3, DLT, SCR, LAS, SCL4/7, SHR, PAT1, Os19 and LISCL according to the previous classification of GRAS families. The *GRAS* genes were very unevenly distributed in different subfamilies. For example, the LISCL subfamily containing 37 GRAS members formed the largest subfamily, including 20 *I. trifida GRAS* genes, seven *Arabidopsis GRAS* genes, and 10 rice *GRAS* genes, whereas the LAS, Os19 and SCL4/7 subfamilies were the relatively small subfamilies, and most of them contained only 3–5 GRAS members. Notably, only one *GRAS* gene in the DLT subfamily was found in those three species. The number of *ItfGRAS* genes was approximately 10 in the HAM, SHR and PAT1 subfamilies, whereas four and six were found in the SCL3 and DELLA subfamilies, respectively.

### Gene structure analyses

**Fig. 3:**
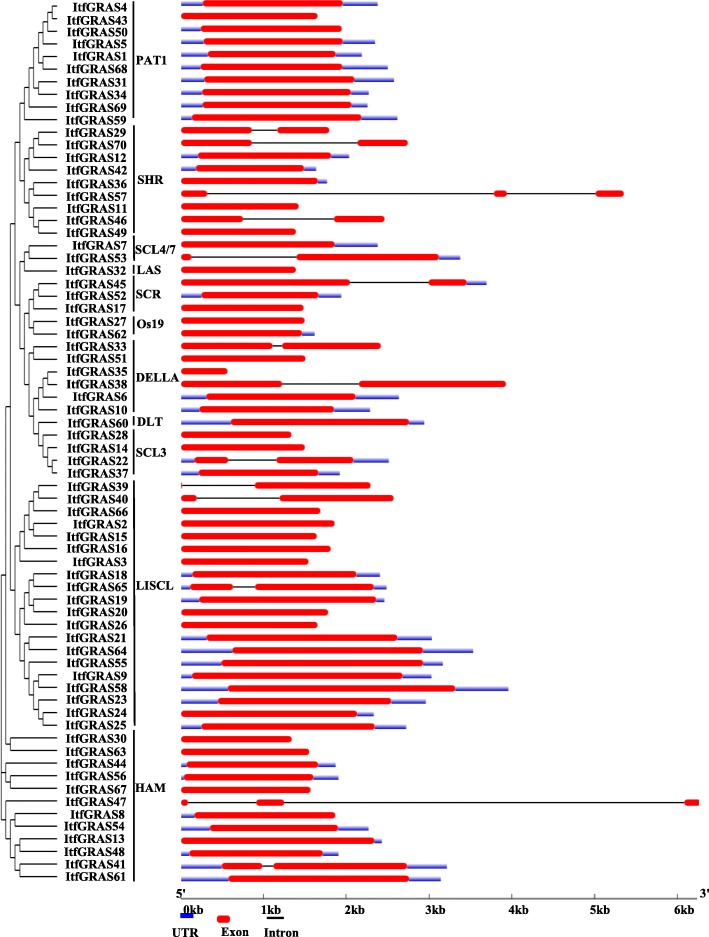
Gene structure of GRAS members in *I. trifida*. The phylogenetic tree of *ItfGRAS* genes is shown on the left, which was divided into 11 clusters, including PAT1, SHR, SCL4/7, LAS, SCR, Os19, DELLA, DLT, SCL3, LISCL and HAM. Schematic diagram of exon/intron structure was displayed by the gene structure display server (GSDS) (http://gsds.cbi.pku.edu.cn/). The exons, introns and UTR are represented by red solid boxes, black lines and blue boxes, respectively

To evaluate the likely diversity of GRAS transcription factors, we conducted an exon/intron analysis based on the sequence alignment between coding sequences and genomic sequences for each *I. trifida GRAS* gene (Fig. 3). Results showed that nearly 56 (80%) *ItfGRAS* transcription factors were intronless, which was consistent with previous reports, and only 14 of the 70 *I. trifida GRAS* genes had 1–2 introns. Among the genes, 12 contained just one intron, and two genes (*ItfGRAS47* and *ItfGRAS57*) had two introns. Furthermore, the majority of *GRAS* genes in the same clade generally presented similar gene structures. Nevertheless, some GRAS transcription factors had exceptions in the same clade but with different gene structure, such as *ItfGRAS46* and *ItfGRAS57* in the clade SHR, and *ItfGRAS7* and *ItfGRAS53* in the clade SCL4/7.

### Stress-related *cis*-elements in *ItfGRAS* promoters

**Fig. 4:**
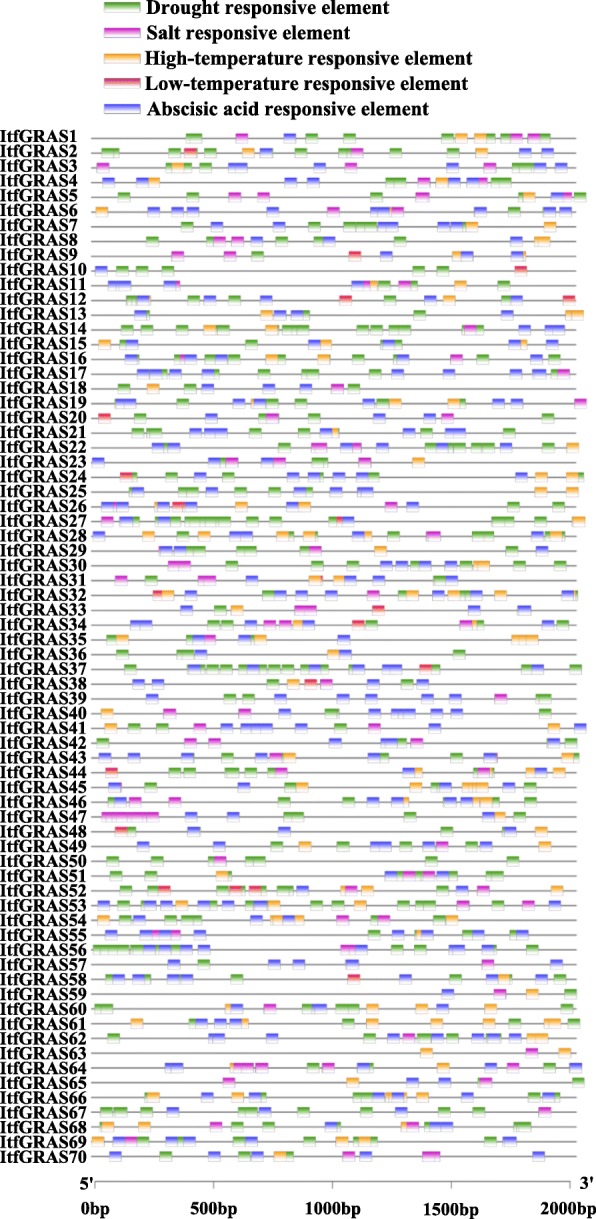
Predicted *cis*-elements in *ItfGRAS* promoters. Promoter sequences (− 2 kb) of 70 *ItfGRAS* genes are analyzed by PlantCARE. Rectangles with different colors indicate that different *cis*-elements participating in various abiotic stress regulation. Green, pink, orange and red bars indicate drought, salt, low- and high-temperaure responsive elements, respectively. And blue bar represents abscisic acid responsive element

In order to further investigate the potential regulatory mechanisms of the *ItfGRASs* under abiotic stress, we obtained 2 kb upstream sequences from the translation initiation site of *ItfGRAS*s and analyzed the *cis*-elements using online tool PlantCARE. Figure 4 showed all predicted different *cis*-elements in the promoter regions of *ItfGRAS*. The results showed that different *cis*-elements participated in various abiotic stresses and hormone responses (Additional file 4: Table S3). *ItfGRASs* excpect *ItfGRAS63*, contained more than one drought responsive elements (MBS, TC-rich repeats, MYB, DRE), indicating that they were involved in drought stress response (Fig. 4). Most *ItfGRASs* (85.7%) contained STRE element, which were associated with high-temperature stress response. About a quarter of these genes have LTR *cis*-element, implying that they might respond to cold stress. 27% of genes, such as *ItfGRAS1*, *ItfGRAS9*, and *ItfGRAS20*, etc., contained GT1-motif elements which were involved in salt stress response. In addition, the *cis*-acting regulatory element MYC found in 97.1% of *ItfGRASs* is related with drought early response and abscisic acid induction. And the drought as well as salt response element DRE was found in 18.6% of *ItfGRSs*, suggesting that these *ItfGRASs* may respond to both drought and salt stresses.

### Expression profile of *ItfGRAS* among various tissues

**Fig. 5:**
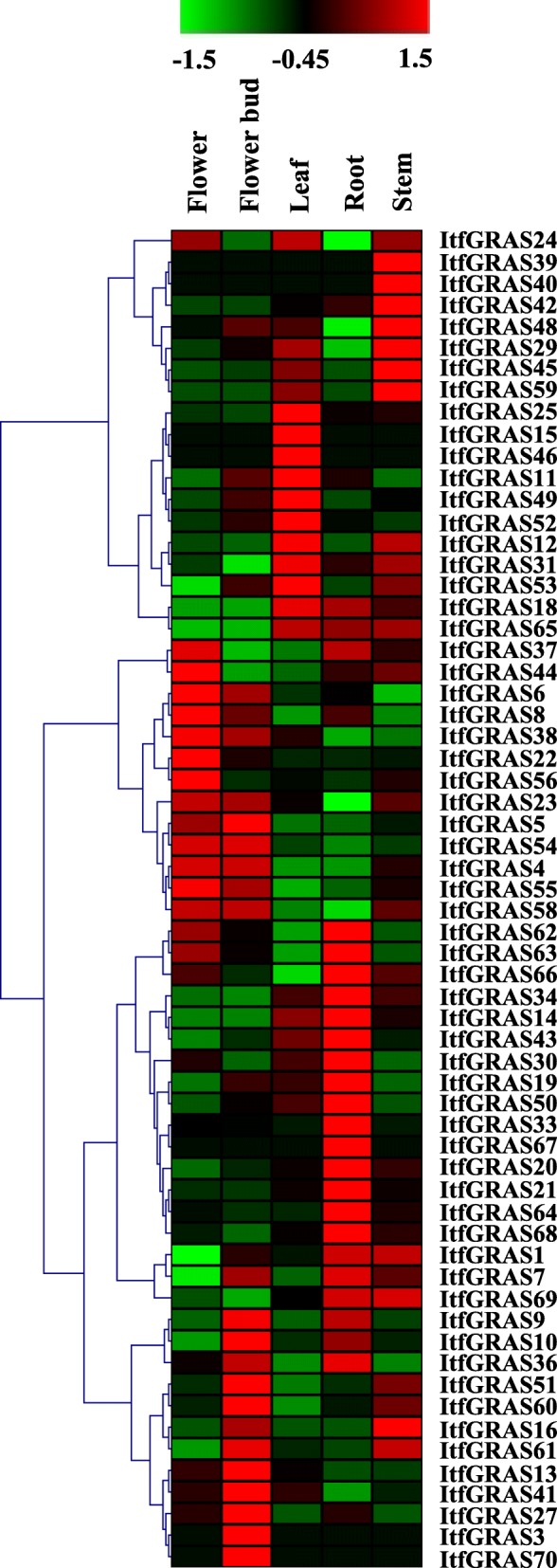
Expression profile of *ItfGRAS* genes among different tissues using RNA-seq. The FPKM values normalized by Z-score are used to measure the expression levels of ItfGRAS transcription factors among various tissues. These tissues include the root, stem, flower, flower bud and leaf. The coloured scale varying from green to red indicates relatively low or high expression. The values of these *GRAS* genes are listed in Additional file 5: Table S4

Increasing evidence of the key role of *GRAS* genes in plant development are available. To investigate the biological functions of *GRAS* genes during different developmental stages, we analysed the transcript levels of *GRAS* genes in different tissues from the root, stem, leaf, flower and flower bud by using public data. A heatmap was generated, which exhibited the expression pattern of ItfGRAS transcription factors among five tissues based on the FPKM values normalized by Z-score (Fig. 5 and Additional file 5: Table S4). Among the *ItfGRAS* genes detected from RNA-seq, 14 (22.3%) *GRAS* genes had relatively higher levels across five tissues, whereas 15 (24.2%) *GRAS* genes were expressed at very low levels among these tissues. Nevertheless, some GRAS transcripts exhibited tissue-specific. For instance, *ItfGRAS7* and *ItfGRAS43* had a low expression in flower relative to those detected in the other tissues. Four *GRAS* genes (*ItfGRAS12*, *ItfGRAS45* and *ItfGRAS59*) were expressed at higher levels in the leaf and stem than in the other tissues, except that *ItfGRAS12* had no change in the flower. In addition, 28 (45.2%) and 34 (54.8%) *GRAS* genes were relatively highly expressed in the root and stem, respectively. Results suggested that the functions of *ItfGRAS* genes greatly changed in different tissues.

### Responses of *ItfGRAS* genes to different stress treatments

**Fig. 6:**
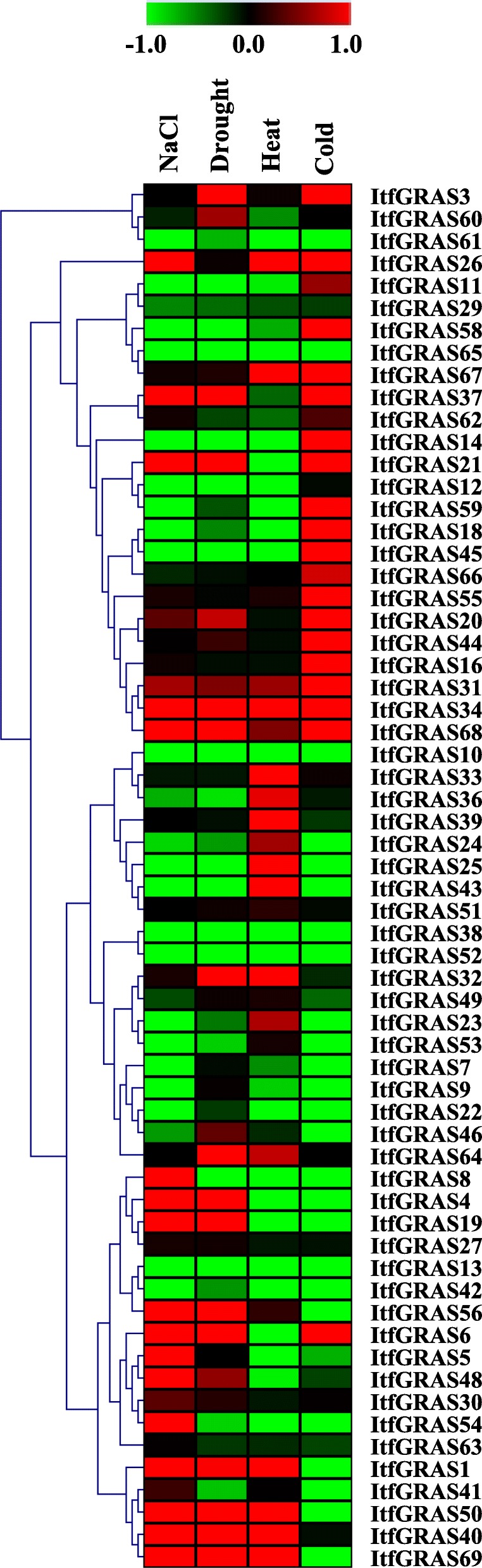
Expression analysis of *ItfGRAS* gene transcript levels under abiotic stresses. FPKM values were used to measure the expression levels of *ItfGRAS* genes in Drought, NaCl, Cold and Heat treatments. Green and red scales indicate relatively low or high expression, respectively. The FPKM values of abiotic stresses for GRAS genes are listed in Additional file 6: Table S5

To survey the possible role of ItfGRAS transcription factors during stress responses, we constructed the heatmap to show the expression profiles of *ItfGRAS* under various stress conditions (Fig. 6). Under four abiotic stresses, more than 15 *ItfGRAS* genes were expressed at relatively high levels, and the number of genes up-regulated in drought stress reached 20. Figure 6 shows that three genes (*ItfGRAS31*, *ItfGRAS34* and *ItfGRAS68*) were all highly expressed under four abiotic stresses. In addition, some *GRAS* genes with high expression levels were found under three abiotic stresses but with low expression under another stress. For instance, *ItfGRAS1*, *ItfGRAS50*, *ItfGRAS56* and *ItfGRAS69* were significantly up-regulated under salt, drought and heat stresses but were down-regulated under cold stress. *ItfGRAS6*, *ItfGRAS20*, *ItfGRAS21* and *ItfGRAS37* were significantly up-regulated during drought, salt and cold stresses but were down-regulated under heat stress treatment. Some transcripts were certainly up-regulated under one stress condition but were down-regulated under other the three stress conditions (*ItfGRAS8*, *ItfGRAS11*, *ItfGRAS23*, *ItfGRAS24*, *ItfGRAS25*, *ItfGRAS36*, *ItfGRAS43*, *ItfGRAS46*, *ItfGRAS54* and *ItfGRAS58*).

### Confirmation of transcriptome data by qRT-PCR in different tissues and under various abiotic stress conditions

**Fig. 7:**
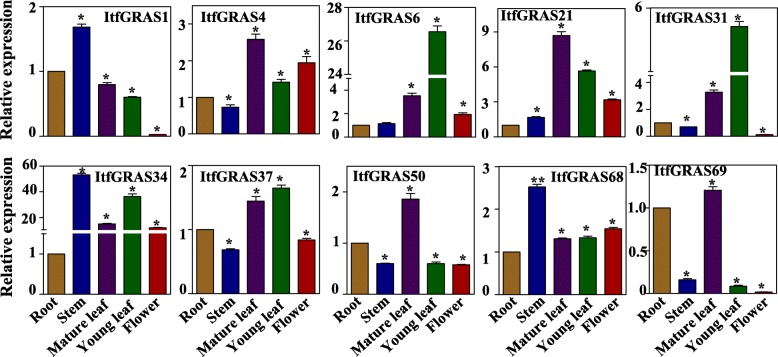
Tissue-specific expression patterns of 10 selected *ItfGRAS* genes by real-time quantitative RT-PCR analysis. The y-axis stands for the relative expression of *ItfGRAS* genes. The x-axis represents different tissues including the root, stem, flower, mature leaf (ML), and young leaf (YL). The expression level is relative to root sample (1). GAPDH was used as an interval control, and standard errors (bar) stand for standard deviations for three replicates. The asterisk indicates that the expression level between other tissues and root is significantly different from the control values (*P* < 0.05)

To further confirm the validity of transcriptome data, we conducted qRT-PCR to check expression pattern of 10 *ItfGRAS* genes (*ItfGRAS1*, *ItfGRAS4*, *ItfGRAS6*, *ItfGRAS21*, *ItfGRAS31*, *ItfGRAS34*, *ItfGRAS37*, *ItfGRAS50*, *ItfGRAS68* and *ItfGRAS69*)*.* We designed primers for these 10 selected genes (Additional file 7: Table S6) and firstly investigated their expression profiles among different tissues (root, stem, mature leaf, young leaf and flower). Some of the qRT-PCR results were not consistent with RNA-seq data, which may cause by the false positive effect of transcriptome. However, the results show that *ItfGRAS6*, *ItfGRAS34*, *ItfGRAS37* and *ItfGRAS68* exhibited a relatively high level across these tissues (Fig. 7). The expression levels for *GRAS* genes vary widely among different tissues. For instance, *ItfGRAS21* and *ItfGRAS50* were expressed at low levels in flower but were highly expressed in other three tissues, except that *ItfGRAS50* was low in stem. *ItfGRAS1* and *ItfGRAS31* were weakly expressed in the flower but were highly expressed in other tissues. *ItfGRAS69* showed relatively higher expression levels in root and mature leaf than in the other tissues. Finally, the expression levels of the selected four genes (*ItfGRAS6*, *ItfGRAS21*, *ItfGRAS31* and *ItfGRAS34*) among these tissues were all higher than those of the other genes.

**Fig. 8:**
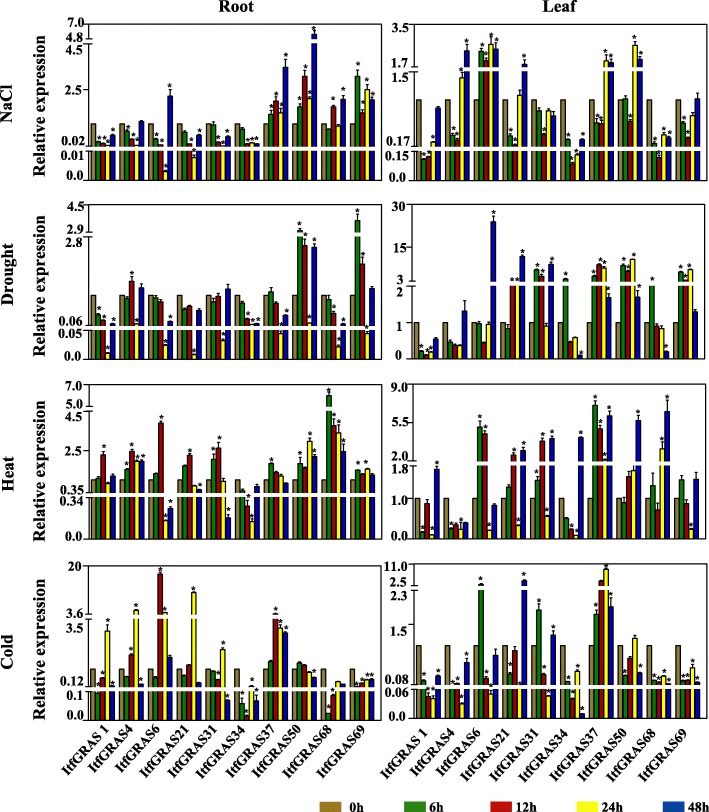
Relative expression levels of 10 selected *ItfGRAS* genes under abiotic stresses by qRT-PCR. The gene expression patterns of root and leaf of *I. trifida* plant were analysed at five time points (0, 6, 12, 24, 48 h) under four stress conditions (cold, heat, drought, and salt). The expression level at 0 h as control was normalised to 1. The expression level of control samples was relative to root sample (1). The error bars show the standard error with three biological replicates. The asterisk indicates a significant change of expression level between the other time period and control at *p* < 0.05

In addition to the analysis of tissue specific expression pattern, the gene responses to abiotic stresses were also checked by qRT-PCR. We analysed the transcription levels of 10 selected *ItfGRAS* genes under salt, drought, cold and heat stress (Fig. 8). Under salt stress treatment, five genes (*ItfGRAS6*, *ItfGRAS37*, *ItfGRAS50*, *ItfGRAS68* and *ItfGRAS69*) were significantly up-regulated in the root, while another *ItfGRAS4* expression was slightly higher at 0 h. In the remaining genes, four genes (*ItfGRAS1*, *ItfGRAS21*, *ItfGRAS31* and *ItfGRAS34*) were clearly down-regulated in the root. The expression of genes in leaves was basically the same as that in the roots under salt stress, except for the up-regulated expression of *ItfGRAS21* and down-regulated expression of *ItfGRAS68* and the substantially unchanged expression level of *ItfGRAS69* compared with that at 0 h. For drought stress in the root, the expression of four GRAS members (*ItfGRAS4*, *ItfGRAS31*, *ItfGRAS50* and *ItfGRAS69*) were up-regulated and that of the other six genes were obviously down-regulated with the lowest expression level at 24 h. Three other significantly up-regulated genes, namely *ItfGRAS6*, *ItfGRAS21* and *ItfGRAS37*, were found in the leaf compared with drought stress in root. After heat treatment, five genes were up-regulated in the root, among which, *ItfGRAS4*, *ItfGRAS50* and *ItfGRAS68* exhibited an obvious increase. The rest of the genes (*ItfGRAS6*, *ItfGRAS21*, *ItfGRAS31*, *ItfGRAS34* and *ItfGRAS37*) were down-regulated, including *ItfGRAS21*, *ItfGRAS31* and *ItfGRAS37* that had the lowest expression levels at 48 h. The expression levels of almost all genes were up-regulated in the leaf with the highest expression levels at 48 h. Only two genes, *ItfGRAS4* and *ItfGRAS6*, were down-regulated in the leaf. Under cold treatment, most genes were down-regulated either in the root or leaf. Among which, the expression levels of four genes (*ItfGRAS1*, *ItfGRAS4*, *ItfGRAS21* and *ItfGRAS31*) in the root increased at 24 h but decreased sharply at 48 h. Three genes (*ItfGRAS1*, *ItfGRAS4* and *ItfGRAS6*) had the lowest expression levels at 24 h in the leaf, and the remaining genes (*ItfGRAS34*, *ItfGRAS50*, *ItfGRAS68* and *ItfGRAS69*) showed the lowest expression levels at 48 h. In addition, two genes (*ItfGRAS6* and *ItfGRAS37* in root; *ItfGRAS21* and *ItfGRAS37* in leaf) were markedly up-regulated under cold stress, whereas the expression level of another gene, *ItfGRAS31*, was only relatively high in the leaf.

## Discussion

The GRAS family is an important plant-specific transcriptional regulator that plays essential roles in regulating plant growth, development and stress responses. In recent years, with the rapid development of bioinformatics analysis, reports on the whole-genome identification of GRAS transcription factors in plants increased. So far, the characteristics of GRAS transcription factors in *I. trifida* remain unclear. Thus, we performed a comprehensive analysis of GRAS transcription factors in *I. trifida* genome, including their phylogenetic relationships, chromosome distribution and gene structure. We also analysed tissue-specific expression patterns and expression profiles in response to stresses.

In this investigation, 70 GRAS transcription factors in *I. trifida* were identified in total, which is higher than the number of *A. thaliana* (33) [4], *Jatropha curcas* (48) [29], *P. mume* (46) [9] and Chinese cabbage (46) [7] but lower than that in other species such as maize (86) [30] and *Populus euphratica* (106) [10]. The number of *GRAS* genes in *I. trifida* is larger, probably because its genome (462.0 Mb) is greater than those of *A. thaliana* (125.0 Mb), *P. mume* (280.0 Mb), and *Brassica rapa* (283.3 Mb). *ItfGRAS* genes were unevenly distributed on the 15 chromosomes of *I. trifida* with the most members (10) on chr4 and chr5 and relatively few members (2) on chr2, chr8, chr10 and chr15. Functions are more similar among diverse species when the proteins have high sequence similarities [31]. Phylogenetic analysis of GRAS proteins in *I. trifida*, *Arabidopsis* and rice was carried out to construct a phylogenetic tree with 11 major branches. Among these subfamilies, some *I. trifida* GRAS proteins are located in the same clade with that of *Arabidopsis* or rice, suggesting their similar functions among different species.

The overall pattern of intron position plays vital roles in the evolution of transcription factor families [32]. Most *ItfGRAS* genes lack introns (Fig. 3) similar to those in some species such as *Arabidopsis*, *Medicago truncatula*, *P. mume*, tomato and *Populus* [4, 7–9, 33]. These results suggested a close evolutionary relationship among GRAS proteins. Intronless genes are also found in the F-box transcription factor gene family [34] and DEAD box RNA helicase [35]. In addition, most GRAS members have similar exon–intron structure, indicating that the structures of *GRAS* genes are highly conserved.

The expression patterns of GRAS transcription factors differed across various tissues, consistent with those reported in other species such as tomato [8] and grapevine [36]. DELLA genes act as the major signalling hub for regulating plant growth and development processes [13, 37]. The three genes (*ItfGRAS6*, *ItfGRAS10* and *ItfGRAS38*) of the DELLA subfamily were also highly expressed in multiple tissues, indicating their important roles in controlling various growth and development processes. *ItfGRAS60* belonging to the DLT subfamily has a relatively higher expression in the bud than the other tissues, suggesting the function of this gene in bud development. All *ItfGRAS* genes in the PAT1 subfamily were highly expressed in the leaf except *ItfGRAS4* and *ItfGRAS5*; this condition may be related to *AtPAT1*, *AtSCL13* and *AtSCL21* (the close evolutionary relationships to *ItfGRAS* genes in this subfamily) that are involved in phytochrome signal transduction [38].

GRAS members are related to regulating their biochemical activities in response to abiotic stresses [39]. Here, most *ItfGRAS* genes were influenced by various stress conditions, except that *ItfGRAS3*, *ItfGRAS26* and *ItfGRAS40* had no significant change under four abiotic stresses. We also analysed the expression of the selected *ItfGRAS* genes under four abiotic stresses with qRT-PCR, which was similar to the result of RNA-seq data. However, the expression levels of several genes from qRT-PCR showed opposite results compared with the public data. For instance, *ItfGRAS34* was expressed at low levels in salt, drought and cold treatments in the qRT-PCR results, but public data showed their high expression under these stresses. DELLA protein plays an important role in regulating plant stress tolerance [40]. One of DELLA members, *ItfGRAS6,*was up-regulated under the salt, drought and cold treatments but was down-regulated under heat condition. By contrast, another DELLA member *ItfGRAS51* was only up-regulated under heat treatment. In addition, Park et al. (2013) proved the significant up-regulation of *BoGRAS* gene during heat stress condition in *Brassica oleracea* [41]. All ItfGRAS members in the PAT1 subfamily were highly expressed under at least one abiotic stress. Among which, four members (*ItfGRAS31*, *ItfGRAS34*, *ItfGRAS68* and *ItfGRAS69*) showed response to four abiotic stresses, suggesting their vital roles in response to adversity stresses, which is consistent with the report on *VaPAT1* [42]. Overall, *I. trifida GRAS* genes have potential regulatory roles in plant development and response to adverse environmental stresses.

## Conclusions

In this study, we identified 70 *GRAS* genes from *I. trifida* which is the closest relative of sweet potato. These *GRAS* genes were distributed on 15 chromosomes and divided into 11 subfamilies. The most genes lack introns and presented similar intron-exon structures, suggesting that the structures of *GRAS* gene were highly conserved. The stress-related *cis*-element analysis, RNA-Seq data and qRT-PCR results indicated the potential functions of ItfGRASs during plant development and stress responses. Our findings will further facilitate the functional study of *GRAS* genes and molecular breeding of sweet potato.

## Supplementary information

**Additional file 1: Fig. S1.** Multiple sequence alignment of 70 *ItfGRAS* genes. The most conserved motif of VHIID is underlined with a black solid line. (PPT 1462 kb)

**Additional file 2: Table S1.** Detailed information of the ItfGRAS. (XLS 29 kb)

**Additional file 3: Table S2.** *Arabidopsis* and rice *GRAS* genes used for phylogenetic analysis. (XLS 26 kb)

**Additional file 4: Table S3.** *Cis*-elements associated with abiotic stresses within the *ItfGRAS* gene promoters.

**Additional file 5: Table S4.** The expression data of the *ItfGRAS* genes in various tissues. (XLS 32 kb)

**Additional file 6: Table S5.** The expression data of the *GRAS* genes under four abiotic stress conditions in *I.trifida. (XLS 33 kb)*

**Additional file 7: Table S6.** Gene-specific primers used for qRT-PCR analysis of the *ItfGRAS* genes. (XLS 20 kb)

## Data Availability

All data generated or analysed during this study are included in this published article and its supplementary information files.

## Abbreviations

AA: Amino acid
FPKM: fragments per kilobase million
GSDS: Gene structure display server
MW: Molecular weights
NJ: Neighbour-joining
pI: Isoelectric points
qRT-PCR: Quantitative reverse transcription polymerase chain reaction
SMART: Simple Modular Architecture Research Tool

## References

[1] Pysh LD, Wysocka-Diller JW, Camilleri C, Bouchez D, Benfey PN (2010). The *GRAS* gene family in arabidopsis: sequence characterization and basic expression analysis of the *SCARECROW-LIKE* genes. Plant J.

[2] Bolle C (2004). The role of GRAS proteins in plant signal transduction and development. Planta..

[3] Sun X, Xue B, Jones WT, Rikkerink E, Dunker AK, Uversky VN (2011). A functionally required unfoldome from the plant kingdom: intrinsically disordered N-terminal domains of GRAS proteins are involved in molecular recognition during plant development. Plant Mol Biol.

[4] Tian C, Wan P, Sun S, Li J, Chen M (2004). Genome-wide analysis of the *GRAS* gene family in rice and *Arabidopsis*. Plant Mol Biol.

[5] Hirsch S, Oldroyd GED (2009). GRAS-domain transcription factors that regulate plant development. Plant Signal Behav.

[6] Zhang J, Zhao Y, Xiao H, Zheng Y, Yue B (2014). Genome-wide identification, evolution, and expression analysis of RNA-binding glycine-rich protein family in maize. J Integr Plant Biol.

[7] Song X, Liu T, Duan W, Ma Q, Ren J, Wang Z (2014). Genome-wide analysis of the *GRAS* gene family in Chinese cabbage (*Brassica rapa* ssp. pekinensis). Genomics..

[8] Huang W, Xian Z, Kang X, Tang N, Li Z (2015). Genome-wide identification, phylogeny and expression analysis of *GRAS* gene family in tomato. BMC Plant Biol.

[9] Lu J, Wang T, Xu Z, Sun L, Zhang Q (2015). Genome-wide analysis of the *GRAS* gene family in *Prunus mume*. Mol Gen Genomics.

[10] Liu X, Widmer A (2014). Genome-wide comparative analysis of the *GRAS* gene family in *populus*, *Arabidopsis* and rice. Plant Mol Biol Rep.

[11] Mayrose M, Ekengren SK, Melech-Bonfil S, Martin GB, Sessa G (2006). A novel link between tomato *GRAS* genes, plant disease resistance and mechanical stress response. Mol Plant Pathol.

[12] Morohashi K, Minami M, Takase H, Hotta Y, Hiratsuka K (2003). Isolation and characterization of a novel *GRAS* gene that regulates meiosis-associated gene expression. J Biol Chem.

[13] Bolle C (2016). Chapter 19-functional aspects of GRAS family proteins. Plant Transcr Factors.

[14] Xu K, Chen S, Li T, Ma X, Liu X, Luo L (2015). *OsGRAS23*, a rice GRAS transcription factor gene, is involved in drought stress response through regulating expression of stress-responsive genes. BMC Plant Biol.

[15] Ma H, Liang D, Shuai P, Xia X, Yin W (2010). The salt- and drought-inducible poplar GRAS protein SCL7 confers salt and drought tolerance in *Arabidopsis thaliana*. J Exp Bot.

[16] Yang M, Yang Q, Fu T, Zhou Y (2011). Overexpression of the *Brassica napus BnLAS* gene in *Arabidopsis* affects plant development and increases drought tolerance. Plant Cell Rep.

[17] Achard P, Gong F, Cheminant S, Alioua M, Hedden P, Genschik P (2008). The cold-inducible CBF1 factor-dependent signaling pathway modulates the accumulation of the growth-repressing DELLA proteins via its effect on gibberellin metabolism. Plant Cell.

[18] Jiang C, Gao X, Liao L, Harberd NP, Fu X (2007). Phosphate starvation root architecture and anthocyanin accumulation responses are modulated by the gibberellin- DELLA signaling pathway in *Arabidopsis*. Plant Physiol.

[19] Czikkel BE, Maxwell DP (2007). *NtGRAS1*, a novel stress-induced member of the GRAS family in tobacco, localizes to the nucleus. J Plant Physiol.

[20] Liu Q (2017). Improvement for agronomically important traits by gene engineering in sweet potato. Breed Sci.

[21] Munoz-Rodriguez P, Carruthers T, Wood JRI, Williams BRM, Weitemier K, Kronmiller B (2018). Reconciling conflicting phylogenies in the origin of sweet potato and dispersal to Polynesia. Curr Biol.

[22] Roullier C, Duputié A, Wennekes P, Benoit L, Bringas VMF, Rossel G (2013). Disentangling the origins of cultivated sweet potato (*Ipomoea batatas (L.) lam*.). PLoS One.

[23] Lu Y, Sun J, Yang Z, Zhao C, Zhu M, Ma D (2019). Genome-wide identification and expression analysis of glycine-rich RNA-binding protein family in sweet potato wild relative *Ipomoea trifida*. Gene.

[24] Guo A, Zhu Q, Chen X, Luo J (2007). GSDS: a gene structure display server. Hereditas..

[25] Kumar S, Stecher G, Tamura K (2016). MEGA 7: molecular evolutionary genetics analysis version 7.0 for bigger datasets. Mol Biol Evol.

[26] Lescot M, Déhais P, Thijs G, Marchal K, Moreau Y, Rouzé P (2002). PlantCARE, a database of plant cis-acting regulatory elementsand a portal to tools for in silico analysis of promoter sequences. Nucleic Acids Res.

[27] Saeed AI, Sharov V, White J, Li J, Liang W, Bhagabati N (2003). TM4: a free, open-source system for microarray data management and analysis. Biotechniques..

[28] Livak KJ, Schmittgen TD (2001). Analysis of relative gene expression data using real-time quantitative PCR and the 2DDCt method. Methods.

[29] Wu Z, Wu P, Chen Y, Li M, Wu G, Jiang H (2015). Genome-wide analysis of the *GRAS* gene family in physic nut (*Jatropha curcas L*.). Genet Mol Res.

[30] Guo Y, We H, Li X, Li Q, Zhao X, Duan X (2017). Identification and expression of GRAS family genes in maize (*Zea mays L.*). PLoS One.

[31] Chen F, Mackey AJ, Vermunt JK, Roos DS (2007). Assessing performance of orthology detection strategies applied to eukaryotic genomes. PLoS One.

[32] Bai Y, Meng Y, Huang D, Qi Y, Chen M (2011). Origin and evolutionary analysis of the plant-specific TIFY transcription factor family. Genomics.

[33] Zhang H, Cao Y, Shang C, Lie J, Wang J, Wu Z (2017). Genome-wide characterization of GRAS family genes in Medicago truncatula reveals their evolutionary dynamics and functional diversification. PLoS One.

[34] Jain M, Nijhawan A, Arora R, Agarwal P, Ray S, Sharma P (2007). F-box proteins in rice. Genome-wide analysis, classification, temporal and spatial gene expression during panicle and seed development, and regulation by light and abiotic stress. Plant Physiol.

[35] Aubourg S, Kreis M, Lecharny A (1999). The DEAD box RNA helicase family in *Arabidopsis thaliana*. Nucleic Acids Res.

[36] Grimplet J, Agudelo-Romero P, Teixeira RT, Martinez-Zapater JM, Fortes AM (2016). Structural and functional analysis of the *GRAS* gene family in grapevine indicates a role of GRAS proteins in the control of development and stress responses. Front Plant Sci.

[37] Wild M, Daviere JM, Cheminant S, Regnault T, Baumberger N, Heintz D (2012). The *Arabidopsis* DELLA RGA-LIKE3 is a direct target of MYC2 and modulates jasmonate signaling responses. Plant Cell.

[38] Bolle C, Koncz C, Chua NH (2000). *PAT1*, a new member of the GRAS family, is involved in phytochrome a signal transduction. Genes Dev.

[39] Niu Y, Zhao T, Xu X, Li J (2017). Genome-wide identification and characterization of GRAS transcription factors in tomato (*Solanum lycopersicum*). PeerJ..

[40] Achard P, Genschik P (2009). Releasing the brakes of plant growth: how GAs shutdown DELLA proteins. J Exp Bot.

[41] Park HJ, Jung WY, Lee SS, Song J, Kwon SY, Kim H (2013). Use of heat stress responsive gene expression levels for early selection of heat tolerant cabbage (*Brassica oleracea L.*). Int J Mol Sci.

[42] Yuan Y, Fang L, Karungo SK, Zhang L, Gao Y, Li S (2016). Overexpression of *VaPAT1*, a GRAS transcription factor from *Vitis amurensis*, confers abiotic stress tolerance in *Arabidopsis*. Plant Cell Rep.

