# Supplementary material for: Identification and expression analysis of GRAS transcription factors in the wild relative of sweet potato Ipomoea trifida
Source: BMC Genomics. 2019 Nov 29;20:911. doi: 10.1186/s12864-019-6316-7 (PMC6884806; doi:10.1186/s12864-019-6316-7)
Supplement: Supplementary file 1 — Additional file 1: Fig. S1. Multiple sequence alignment of 70 ItfGRAS genes. The most conserved motif of VHIID is underlined with a black solid line. (PPT 1462 kb) [file 12864_2019_6316_MOESM1_ESM.ppt]

## Slide 1
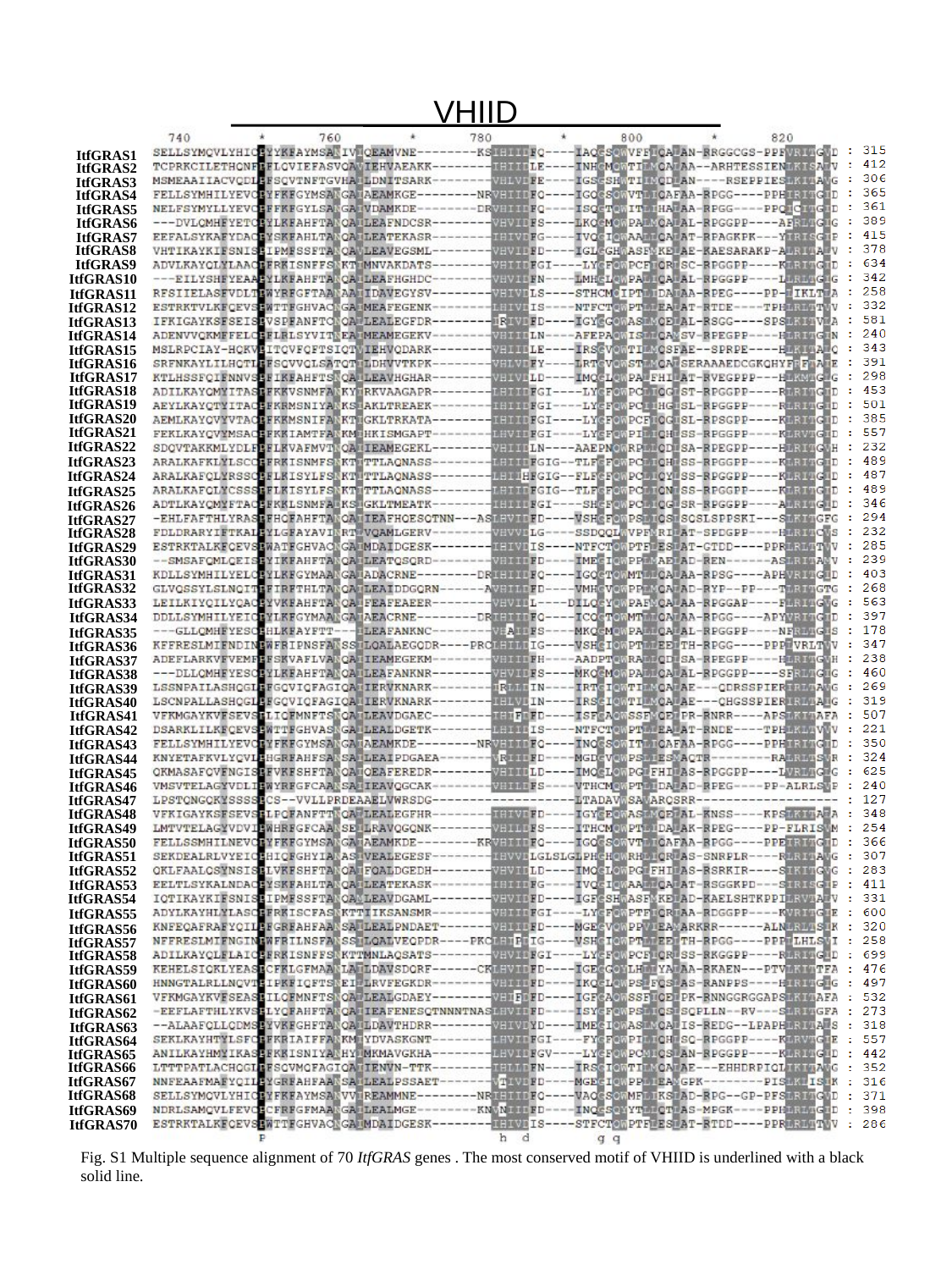

VHIID
ItfGRAS1
ItfGRAS2
ItfGRAS3
ItfGRAS4
ItfGRAS5
ItfGRAS6
ItfGRAS7
ItfGRAS8
ItfGRAS9
ItfGRAS10
ItfGRAS11
ItfGRAS12
ItfGRAS13
ItfGRAS14
ItfGRAS15
ItfGRAS16
ItfGRAS17
ItfGRAS18
ItfGRAS19
ItfGRAS20
ItfGRAS21
ItfGRAS22
ItfGRAS23
ItfGRAS24
ItfGRAS25
ItfGRAS26
ItfGRAS27
ItfGRAS28
ItfGRAS29
ItfGRAS30
ItfGRAS31
ItfGRAS32
ItfGRAS33
ItfGRAS34
ItfGRAS35
ItfGRAS36
ItfGRAS37
ItfGRAS38
ItfGRAS39
ItfGRAS40
ItfGRAS41
ItfGRAS42
ItfGRAS43
ItfGRAS44
ItfGRAS45
ItfGRAS46
ItfGRAS47
ItfGRAS48
ItfGRAS49
ItfGRAS50
ItfGRAS51
ItfGRAS52
ItfGRAS53
ItfGRAS54
ItfGRAS55
ItfGRAS56
ItfGRAS57
ItfGRAS58
ItfGRAS59
ItfGRAS60
ItfGRAS61
ItfGRAS62
ItfGRAS63
ItfGRAS64
ItfGRAS65
ItfGRAS66
ItfGRAS67
ItfGRAS68
ItfGRAS69
ItfGRAS70
Fig. S1 Multiple sequence alignment of 70 ItfGRAS genes . The most conserved motif of VHIID is underlined with a black solid line.
