# Supplementary material for: Identification and expression analysis of GRAS transcription factors in the wild relative of sweet potato Ipomoea trifida
Source: BMC Genomics. 2019 Nov 29;20:911. doi: 10.1186/s12864-019-6316-7 (PMC6884806; doi:10.1186/s12864-019-6316-7)
Supplement: Supplementary file 4 — Additional file 4: Table S3. Cis-elements associated with abiotic stresses within the ItfGRAS gene promoters. [file 12864_2019_6316_MOESM4_ESM.doc]

**Table S3. *Cis*-elements associated with abiotic stresses within the *ItfGRAS* gene promoter.**

| **Gene nam**e | **Drought responsive element** | | | | | **Low-temperature responsive element** | **High-temperature responsive element** | **Salt responsive element** | | **Abscisic acid responsive element** | |
| --- | --- | --- | --- | --- | --- | --- | --- | --- | --- | --- | --- |
|  | MBS | TC-rich repeats | MYB | MYC | DRE | LTR | STRE | GT1-motif | DRE | MYC | ABRE |
| ItfGRAS1 | √ | √ | √ | √ |  |  | √ | √ |  | √ | √ |
| ItfGRAS2 | √ |  | √ | √ | √ | √ | √ |  | √ | √ | √ |
| ItfGRAS3 |  | √ | √ | √ | √ |  | √ | √ | √ | √ | √ |
| ItfGRAS4 | √ |  | √ | √ |  |  | √ | √ |  | √ |  |
| ItfGRAS5 |  |  | √ | √ | √ |  | √ | √ | √ | √ |  |
| ItfGRAS6 |  |  |  | √ |  |  | √ | √ |  | √ | √ |
| ItfGRAS7 |  |  | √ | √ |  |  | √ |  |  | √ | √ |
| ItfGRAS8 |  | √ | √ | √ | √ |  | √ |  | √ | √ | √ |
| ItfGRAS9 | √ |  |  | √ |  | √ | √ | √ |  | √ | √ |
| ItfGRAS10 | √ | √ | √ | √ |  | √ |  |  |  | √ |  |
| ItfGRAS11 | √ |  | √ | √ |  |  | √ | √ |  | √ | √ |
| ItfGRAS12 | √ | √ | √ | √ |  | √ | √ |  |  | √ |  |
| ItfGRAS13 |  |  | √ | √ |  |  | √ |  |  | √ | √ |
| ItfGRAS14 | √ | √ | √ | √ |  |  | √ | √ |  | √ |  |
| ItfGRAS15 | √ |  | √ | √ |  |  | √ |  |  | √ | √ |
| ItfGRAS16 | √ |  | √ | √ |  |  | √ | √ |  | √ |  |
| ItfGRAS17 | √ |  | √ | √ | √ |  |  |  | √ | √ | √ |
| ItfGRAS18 |  |  | √ | √ |  |  | √ | √ |  | √ |  |
| ItfGRAS19 | √ | √ | √ | √ |  |  | √ | √ |  | √ | √ |
| ItfGRAS20 | √ |  | √ | √ |  | √ |  | √ |  | √ | √ |
| ItfGRAS21 |  | √ | √ | √ |  |  | √ |  |  | √ |  |
| ItfGRAS22 | √ |  | √ | √ |  |  | √ | √ |  | √ | √ |
| ItfGRAS23 |  | √ | √ | √ | √ |  | √ | √ | √ | √ | √ |
| ItfGRAS24 |  | √ |  | √ |  | √ | √ |  |  | √ | √ |
| ItfGRAS25 | √ |  | √ | √ |  |  | √ |  |  | √ | √ |
| ItfGRAS26 | √ |  | √ | √ | √ | √ | √ | √ | √ | √ | √ |
| ItfGRAS27 | √ | √ | √ | √ |  | √ | √ |  |  | √ | √ |
| ItfGRAS28 |  |  | √ | √ |  |  | √ | √ |  | √ |  |
| ItfGRAS29 | √ |  | √ | √ |  |  | √ | √ |  | √ |  |
| ItfGRAS30 | √ |  | √ | √ |  |  | √ | √ |  | √ |  |
| ItfGRAS31 |  |  | √ | √ | √ | √ | √ | √ | √ | √ |  |
| ItfGRAS32 |  |  | √ | √ |  | √ | √ | √ |  | √ | √ |
| ItfGRAS33 | √ |  | √ | √ |  | √ | √ | √ |  | √ | √ |
| ItfGRAS34 |  | √ | √ | √ | √ | √ | √ | √ | √ | √ | √ |
| ItfGRAS35 | √ |  | √ | √ | √ |  | √ |  | √ | √ |  |
| ItfGRAS36 | √ |  | √ | √ |  |  | √ |  |  | √ |  |
| ItfGRAS37 | √ |  | √ | √ |  | √ |  |  |  | √ | √ |
| ItfGRAS38 |  |  |  | √ |  | √ | √ | √ |  | √ | √ |
| ItfGRAS39 |  |  | √ | √ |  |  |  | √ |  | √ | √ |
| ItfGRAS40 |  | √ | √ | √ |  |  | √ | √ |  | √ | √ |
| ItfGRAS41 | √ |  | √ | √ | √ |  | √ | √ | √ | √ | √ |
| ItfGRAS42 |  |  |  | √ |  |  |  | √ |  | √ | √ |
| ItfGRAS43 | √ |  | √ | √ |  |  | √ | √ |  | √ | √ |
| ItfGRAS44 | √ | √ | √ | √ |  | √ | √ | √ |  | √ |  |
| ItfGRAS45 | √ |  | √ | √ |  |  | √ | √ |  | √ | √ |
| ItfGRAS46 |  | √ |  | √ | √ |  | √ |  | √ | √ | √ |
| ItfGRAS47 |  | √ | √ | √ | √ |  | √ |  | √ | √ |  |
| ItfGRAS48 | √ | √ | √ | √ |  | √ | √ |  |  | √ |  |
| ItfGRAS49 | √ | √ | √ | √ |  |  | √ | √ |  | √ | √ |
| ItfGRAS50 | √ |  | √ |  |  |  |  | √ |  |  |  |
| ItfGRAS51 | √ |  | √ | √ |  |  | √ | √ |  | √ | √ |
| ItfGRAS52 |  | √ | √ | √ |  | √ | √ |  |  | √ | √ |
| ItfGRAS53 | √ |  | √ | √ |  |  | √ | √ |  | √ | √ |
| ItfGRAS54 | √ | √ | √ | √ |  |  | √ | √ |  | √ | √ |
| ItfGRAS55 |  |  | √ | √ |  |  | √ | √ |  | √ | √ |
| ItfGRAS56 | √ |  | √ | √ |  |  |  | √ |  | √ | √ |
| ItfGRAS57 |  |  | √ | √ |  |  |  | √ |  | √ | √ |
| ItfGRAS58 | √ | √ | √ | √ |  | √ | √ |  |  | √ |  |
| ItfGRAS59 |  |  | √ | √ |  |  | √ | √ |  | √ |  |
| ItfGRAS60 | √ |  | √ | √ |  |  | √ | √ |  | √ | √ |
| ItfGRAS61 |  |  | √ | √ |  |  | √ |  |  | √ |  |
| ItfGRAS62 |  | √ | √ | √ |  |  | √ |  |  | √ | √ |
| ItfGRAS63 |  |  |  |  |  |  | √ | √ |  |  |  |
| ItfGRAS64 |  | √ | √ | √ |  |  | √ | √ |  | √ | √ |
| ItfGRAS65 |  |  | √ | √ |  |  | √ | √ |  | √ |  |
| ItfGRAS66 |  |  | √ | √ |  |  | √ |  |  | √ | √ |
| ItfGRAS67 | √ |  | √ | √ |  |  |  |  |  | √ | √ |
| ItfGRAS68 |  |  | √ | √ |  |  | √ | √ |  | √ |  |
| ItfGRAS69 | √ |  | √ | √ |  |  | √ |  |  | √ |  |
| ItfGRAS70 | √ | √ | √ | √ |  |  | √ | √ |  | √ | √ |
